# The VHL tumor suppressor at the crossroad of protein folding, aggregation, and cancer

**DOI:** 10.1002/1878-0261.70325

**Published:** 2026-09-07

**Authors:** Lara Abad, Silvio Carlo Ermanno Tosatto, Emanuela Leonardi

**Affiliations:** ^1^ Department of Biomedical Sciences University of Padova Italy

**Keywords:** amyloid‐targeted therapy, cancer dormancy, protein aggregation, von Hippel–Lindau

## Abstract

Protein aggregation is no longer viewed only as pathological but as a dynamic and reversible regulatory mechanism in cancer. Within the tumor microenvironment, proteins such as the von Hippel‐Lindau tumor suppressor protein (pVHL) can transition from a folded state to aggregated states. Mutations, environmental stress, and dysfunctional chaperone systems further promote pVHL aggregation. Structural plasticity enables adaptive responses that support protein storage, cell survival, and dormancy, a reversible state promoting drug resistance and cancer recurrence. Targeting protein aggregation with chemical chaperones and amyloid inhibitors could represent a promising therapeutic strategy to rescue the tumor suppressor activity and overcome dormancy‐associated drug resistance. In this review, we address the amyloid aggregation of pVHL, the factors contributing to this behavior, the correlation between protein aggregation and cellular dormancy, and potential therapeutic strategies that bridge protein aggregation and oncology.

## Graphical abstract

Mutations, environmental stress, and chaperone dysfunction can destabilize pVHL, promoting its conversion from the native folded state into amyloid‐like assemblies. This transition may contribute to protein storage, cell dormancy, survival, and drug resistance. Chemical chaperones and amyloid inhibitors may counteract pVHL aggregation, highlighting potential strategies to modulate its pathological consequences.

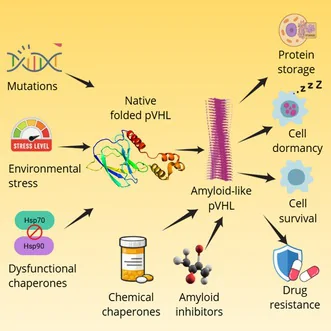

**Abbreviations**
ACM: amyloid‐converting motif
AFM: atomic force microscopy
ccRCC: clear cell renal cell carcinoma
CR: Congo red
Cul 2: Cullin 2
ECV: E3 ubiquitin ligase complex
GSK3: glycogen synthase kinase‐3
HIF: hypoxia‐inducible factor
HIF‐α: hypoxia‐inducible factor alpha
Hsp70: heat shock protein 70
Hsp90: heat shock protein 90
IPOD: insoluble protein deposit
JUNQ: juxtanuclear quality control compartment
LSA: large slow‐moving aggregates
mTOR: mammalian target of rapamycin
NES: nuclear export signal
PCK: protein kinase C
Pfd3: prefoldin subunit 3
PHDs: prolyl hydroxylase domain enzymes
PI3K: phosphoinositide 3‐kinase
pVHL: von Hippel–Lindau tumor suppressor protein
Rbx1: Ring‐Box 1
RCC: renal cell carcinoma
rIGSRNA: ribosomal intergenic noncoding RNA
SDA: small dynamic aggregates
TEM: transmission electron microscopy
TKI: tyrosine kinase inhibitor
TRiC/CCT: TCP‐1 ring complex/chaperonin containing TCP‐1
VCB: VHL‐Elongin C‐Elongin B
VHL: von Hippel–Lindau

## 1 Introduction

Protein folding and aggregation are fundamental molecular processes that extend far beyond their traditional association with neurodegenerative diseases [1, 2]. Increasing evidence reveals that proteins implicated in cancer can also undergo physiological and stress‐induced aggregation, suggesting that this phenomenon is not solely a manifestation of pathology but may also represent a cellular adaptive mechanism [3, 4, 5]. Within the tumor microenvironment, characterized by low oxygen levels, acidosis, and metabolic stress, proteins may shift between folded, disordered, and aggregated states to modulate cellular functions and survival strategies [6]. These physiological amyloids have been correlated, in mammals, with protein synthesis, regulation of kinase activity, and hormone storage, among others [6, 7]. Furthermore, it has been proposed as a mechanism adopted by the cells to regulate protein activity and store them under stress conditions, protecting them from degradation [8].

This dynamic structural plasticity can foster a dormant phenotype, a cellular state in which proliferation is temporarily halted while metabolic activity and cellular viability are preserved [5]. Such dormancy allows tumor cells to endure unfavorable conditions and evade therapies that preferentially target highly proliferating and dividing cells, ultimately contributing to recurrence and drug resistance [5, 9]. Several strategies have been proposed to address cell dormancy and therapy resistance. While some authors advocate maintaining dormancy indefinitely, others suggest that awakening dormant cells to resensitize them to existing therapies may be a more effective approach [5]. However, there is still no consensus on how to manage dormancy in cancer. Thus, understanding how folding and aggregation intersect with cancer biology provides a conceptual bridge between proteostasis and tumor dormancy.

Among the proteins that exemplify this interplay, the von Hippel–Lindau tumor suppressor protein (pVHL) stands out [2]. Its intrinsic molten globule behavior, dependence on molecular chaperones for correct folding, and propensity to form reversible amyloid‐like aggregates place it at the crossroads between protein homeostasis and cancer progression [4, 10]. The best characterized function of pVHL is the regulation of the hypoxia‐inducible factor alfa (HIF‐α) through its ubiquitination and subsequent proteasomal degradation [11, 12]. Under normoxic conditions, prolyl hydroxylase domain enzymes (PHDs) catalyze the hydroxylation of specific proline residues on HIF‐α, enabling its recognition by the β‐domain of pVHL, which targets HIF‐α for proteasomal degradation [12]. Under hypoxic conditions, PHD enzymes are no longer able to hydroxylate HIF‐α, preventing its recognition by pVHL and allowing its escape from degradation. In consequence, HIF‐α translocates to the nucleus where it promotes the transcription of several genes favoring cancer progression [12].

Mutations in the *VHL* gene cause von Hippel–Lindau (VHL) disease, an autosomal dominant cancer syndrome that predisposes individuals to both benign and malignant tumors [12, 13]. VHL disease is classified into four clinical subtypes (types 1, 2A, 2B, and 2C) based on tumor spectrum. Type 1 is characterized by susceptibility to renal cell carcinoma (RCC) and hemangioblastomas but not pheochromocytoma. In contrast, type 2 is associated with pheochromocytoma, with a low (type 2A) or high (type 2B) risk of RCC, whereas type 2C predisposes exclusively to pheochromocytoma [13, 14]. Most clinically significant neoplasms include clear cell renal cell carcinoma (ccRCC), hemangioblastoma, or pheochromocytoma [12, 13, 14].

Since aggressive ccRCC tumors harboring wild‐type (WT) VHL have been reported, there is growing interest in understanding the underlying causes of pVHL inactivation beyond mutations. It has been proposed that pVHL sequestration into amyloid aggregates may simulate loss‐of‐function mutations, contributing to protein inactivation in a mutation‐independent manner [4]. Exploring how the conformational transitions of pVHL from folded to aggregated states affect cellular functions offers an opportunity to reconsider the molecular basis of tumor dormancy and resistance and to identify novel therapeutic strategies targeting protein aggregation in cancer. A detailed understanding of the structural features and folding behavior of pVHL is essential to elucidate how its conformational plasticity governs its function and propensity to aggregate.

## 2 Structural features and folding of pVHL

**Fig. 1:**
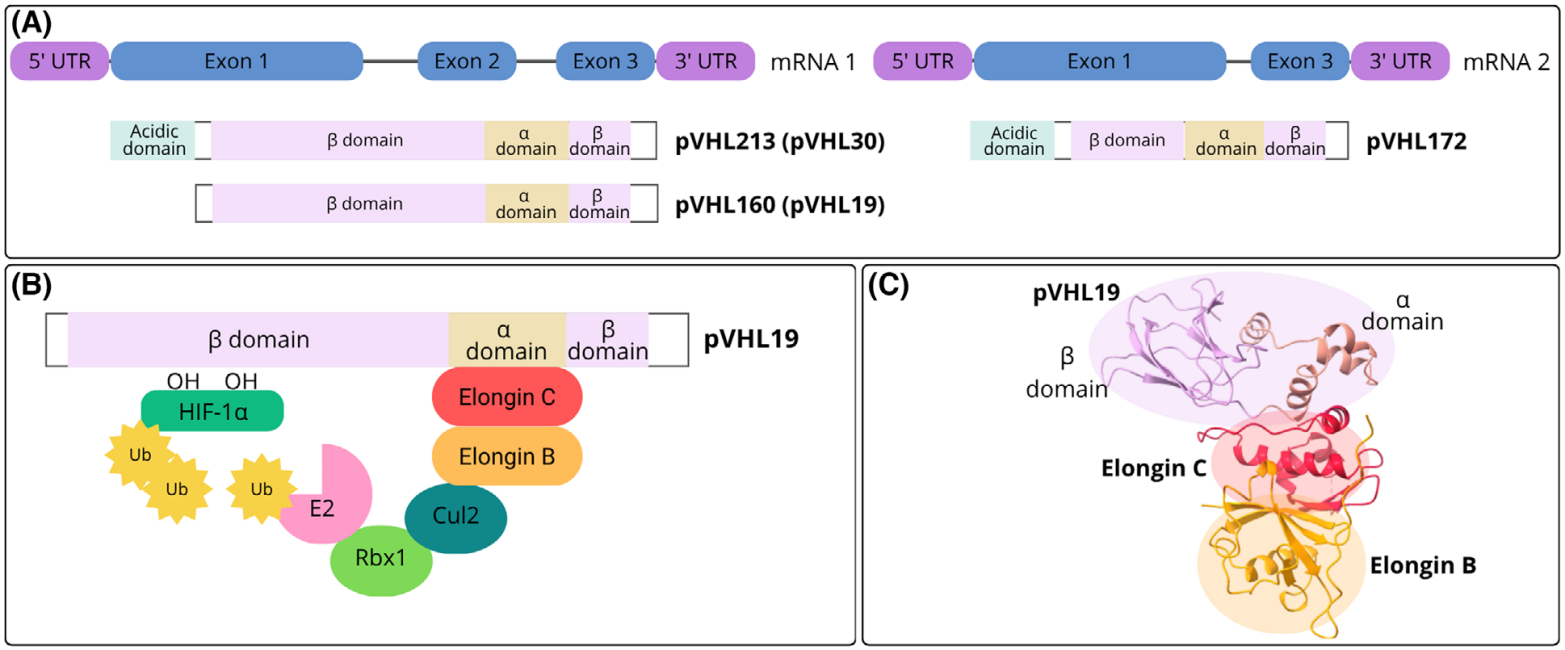
VHL gene organization and VCB complex formation. (A) Three protein isoforms encoded by the human VHL gene from two mRNA transcripts (mRNA_1_ left panel, mRNA_2_ right panel). mRNA_1_ encodes the full‐length protein pVHL213 (pVHL30) and a shorter isoform due to an alternative translation initiation site, pVHL160 (pVHL19). The mRNA_2_ encodes pVHL172, which lacks exon 2. (B) pVHL19 forming the E3 ubiquitin ligase (ECV) complex together with Elongin B, Elongin C, Cul2, and Rbx1. Ubiquitination of the main pVHL's substrate, HIF‐1α. (C) Crystal structure (PDB 1VCB) of pVHL19 in complex with Elongin C and Elongin B. pVHL binds Elongin C through its α‐domain.

The human VHL gene is transcribed into two mRNAs that encode three protein isoforms (Fig. 1A) [13, 15]. The full‐length protein, pVHL213 (also known as pVHL30), and a shorter isoform generated from an alternative translation initiation site, pVHL160 (also known as pVHL19), are translated from mRNA transcript 1 [15, 16]. mRNA transcript 2 encodes pVHL172, which lacks exon 2 [17]. Unlike the latter, pVHL213 (30 kDa) and pVHL160 (19 kDa) possess tumor suppressor activity [15]. Structurally, these two isoforms can be divided into a β‐domain (residues 63–155) and an α‐domain (residues 156–193), with an additional N‐terminal intrinsically disordered region in pVHL213 (acidic domain, residues 1–54) [4, 15, 18]. This structural organization is essential for the role of pVHL as the substrate recognition subunit of the VHL‐Elongin C‐Elongin B (VCB) complex. The β‐domain is responsible for substrate recognition, while the α‐domain interacts with elongin C, which subsequently associates with Cullin 2 (Cul2) followed by Ring‐Box 1 (Rbx1) to assemble the functional E3 ubiquitin ligase complex (ECV) (Fig. 1B) [11, 15, 19].

The crystal structure of pVHL in complex with Elongin C and Elongin B (Fig. 1C) has been experimentally resolved and is available in the Protein Data Bank (PDB code 1VCB) [20]. Other structures present this complex associated with ligands such as HIF‐1α, one of the main substrates of pVHL [21]. So far, no structure has been experimentally resolved for pVHL alone, since when unbound to its interacting partners, it adopts a molten globule conformation, with appreciable secondary structure but a dynamic and fluctuating tertiary structure [16, 22]. Several experimental methods such as size exclusion, light scattering, fluorescence anisotropy, and ultracentrifugation of purified pVHL19 have revealed a non‐compact, monomeric conformation with enlarged hydrodynamic radius and fluorescence features consistent with a partially folded molten globule [22]. Remarkably, while molten globule structures are typically induced under extreme conditions, pVHL displays this conformational state under near‐physiological pH, ionic strength, and temperature, making it one of the few documented examples of a protein adopting such structural plasticity under native conditions [22].

**Fig. 2:**
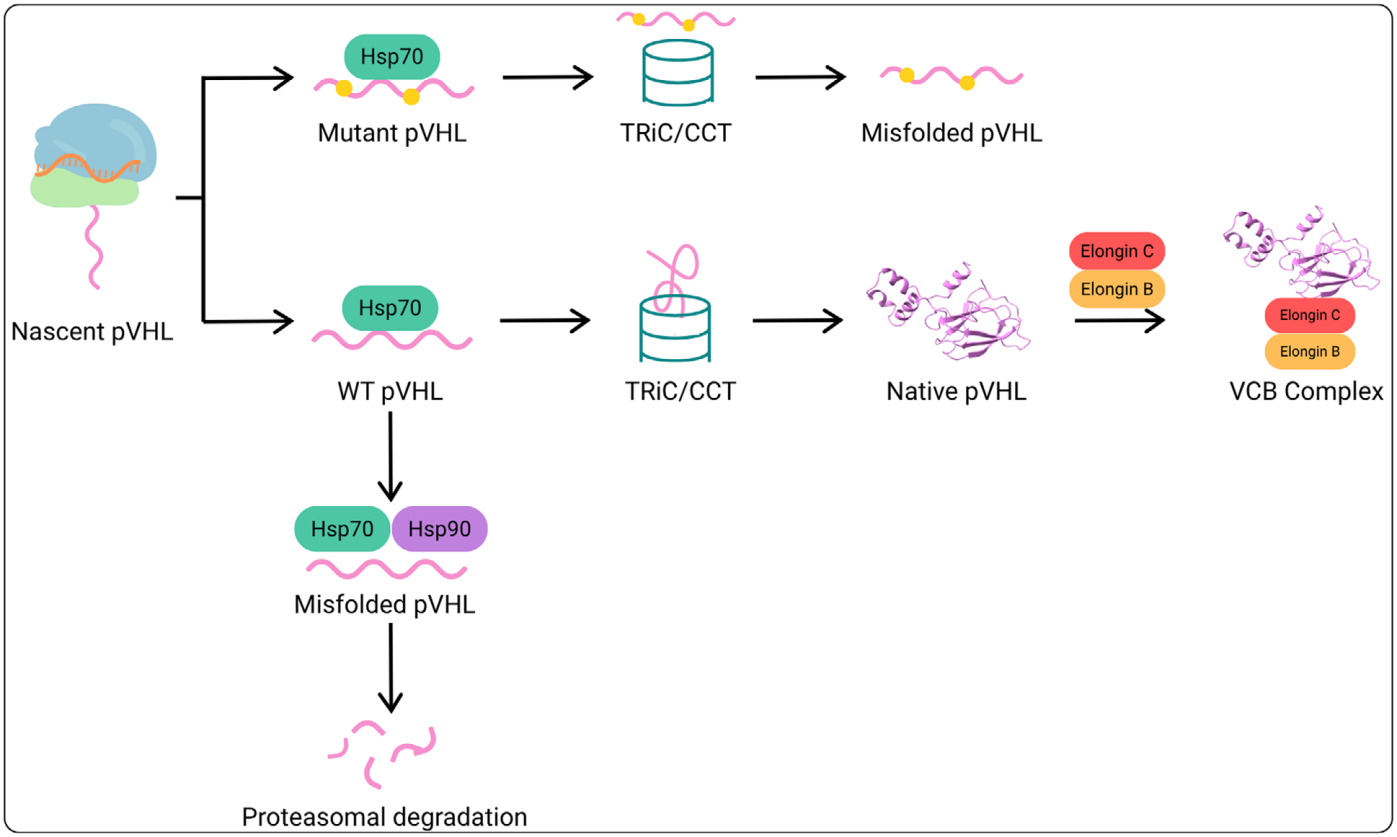
pVHL folding and quality control. Nascent pVHL binds Hsp70, which assists TRiC/CCT in the protein folding process. Misfolded pVHL is recognized by the Hsp70‐Hsp90 quality control system for proteasomal degradation. Native pVHL associates with Elongin C and Elongin B to form the VCB complex. Mutations in pVHL inhibit its binding to the TRiC/CCT complex.

The native folding of pVHL is heavily reliant on molecular chaperones (Fig. 2), a network of proteins that assist newly synthesized polypeptides in reaching their native conformation while preventing inappropriate folding and aggregation [23, 24]. Among these, Hsp70 binds exposed hydrophobic regions of nascent or partially folded proteins to maintain their solubility; the eukaryotic chaperonin TRiC/CCT provides an ATP‐dependent folding chamber for a subset of structurally complex proteins; and Hsp90 contributes to the quality control decision by promoting the maturation or degradation of unstable proteins [23, 24]. Within pVHL, a hydrophobic chaperonin‐binding cluster located roughly between residues 100 and 155 is essential for TRiC/CCT recognition, which facilitates proper tertiary folding and prevents off‐pathway aggregation [10, 24]. Oncogenic mutations in this region such as G114R and I151S destabilize pVHL folding by disrupting its interaction with TRiC, resulting in misfolded intermediates [10, 24, 25]. Although Hsp70 retains the ability to bind the mutated pVHL and preserve its solubility, this interaction alone is insufficient to support assembly of the correctly folded VCB complex [24]. Hsp70 cooperates with TRiC/CCT to enhance folding efficiency, while Hsp90 plays a central role in targeting misfolded or aggregation‐prone pVHL for proteasomal degradation (Fig. 2) [17, 23]. This network creates a finely tuned quality control system in which the structural fate of pVHL (folding vs. degradation) is regulated by the coordinated action of molecular chaperones [23]. Disruption of these pathways, whether by mutations or cellular stress, could lead to accumulation of partially folded or misfolded intermediates that are prone to aggregation [16, 23].

## 3 Misfolding and aggregation propensity of pVHL

Early *in vitro* studies have provided direct evidence of amyloid‐like aggregation of pVHL and peptides corresponding to pVHL fragments [2]. The intrinsic thermodynamic instability of pVHL provides a structural basis for its aggregation propensity [16]. Urea‐induced unfolding experiments revealed that pVHL begins to denature at very low urea concentrations (~ 0.25 m), and this unfolding process was partially irreversible [22]. Moreover, size exclusion chromatography results showed that WT pVHL forms soluble aggregates in addition to monomers, confirming its inherent tendency to self‐associate under native conditions [16].

Several regions within the pVHL sequence have been identified as highly prone to forming β‐sheet‐rich aggregates [2]. *In silico* analyses by Kumar et al. [2] predicted six distinct aggregation‐prone and amyloidogenic segments within the protein: ^72^SQVIFCN^78^, ^85^LPVWLN^90^, ^117^WLF^119^, ^130^VNQTELFVP^138^, ^147^IFANITL^153^, and ^163^LQVVR^167^. Five of these six fragments are located within the β‐domain and encompass four aromatic residues (F76, W117, F119, and F136) that form an aromatic tetrahedron [16]. This tetrahedron links the β strands together, maintaining the β‐sandwich architecture and the overall structure of pVHL [16]. *In vitro*, two of the amyloidogenic fragments (^72^SQVIFCN^78^ and ^147^IFANITL^153^) displayed rapid aggregation kinetics [2]. Transmission electron microscopy (TEM) and atomic force microscopy (AFM) of these aggregates revealed β‐sheet‐rich fibrillar morphology (cross‐β pattern), characteristic hallmarks of amyloid structure [2]. Follow‐up studies demonstrated that a pVHL‐derived peptide (pVHL104‐140) and the full‐length pVHL form amyloid fibrils under *in vitro* physiological‐like conditions as well as under acidic conditions typical of the tumor microenvironment [4]. Kuzmina et al. [1] characterized the fibrils formed by recombinant pVHL30 and GST‐VHL30 under acidic conditions (pH 2, 37 °C) with AFM. The fibril lengths ranged from hundreds of nanometers to several micrometers and showed a hierarchical organization, with multiple protofibrils intertwining into thicker structures. GST‐VHL30 displayed a higher aggregation propensity and produced longer, more uniform fibrils, indicating that the disordered N‐terminal region of pVHL modulates nucleation and fibril growth [1].

Beyond *in vitro* conditions, evidence from living systems further supports the tendency of pVHL to aggregate. As previously mentioned, molecular chaperones play a central role in this process [10, 17]. Interaction between pVHL and Prefoldin is crucial for proper folding into its native structure [15]. In *Schizosaccharomyces pombe*, Prefoldin subunit 3 (Pfd3), originally identified as von Hippel–Lindau binding protein 1, stabilizes human pVHL, prevents its aggregation, and enhances its interaction with HIF‐1α [15]. In this model, pVHL was incorporated into small dynamic (SDA) or large slow‐moving (LSA) aggregates [17]. When expressed in *Saccharomyces cerevisiae*, pVHL30 fails to fold correctly due to the absence of its human cofactors and aggregates in the insoluble protein deposit (IPOD, close to the vacuole) and the juxtanuclear quality control compartment (JUNQ) [17]. In human cell lines (namely HEK293, HeLa, and 786‐O), depletion of PFDN3, the human homolog of Pfd3, similarly destabilizes pVHL [15] and leads to its incorporation into JUNQ [17, 26]. Furthermore, the intrinsic amyloidogenic properties of pVHL were confirmed in a bacterial model where the *E. coli* curli gene was replaced with the VHL gene [4]. pVHL amyloid formation was assessed by Congo red (CR) staining, a specific stain for amyloids that presents apple green birefringence under cross‐polarized light and visualized with transmission electron microscopy (TEM), confirming the intrinsic ability of pVHL to form amyloid fibrils [4].

## 4 Aggregation‐prone VHL mutants

The aggregation propensity of pVHL can be further aggravated by mutations that promote misfolding and stability loss (Table 1) [16, 17, 27]. Although most of VHL mutations map to the HIF‐1α or elongin C interaction site, many others lie outside these regions [20]. This suggests the existence of an alternative mechanism leading to loss of function, where pVHL native folding and conformation are compromised [2, 16, 17]. One of the most frequently mutated residues on the α‐domain is R167, a necessary amino acid for stabilizing the α‐β domain interface [20]. Another frequently mutated residue is L178, also involved in stabilizing the packing of the α‐domain [20]. β‐domain mutations strike the hydrophobic and polar residues that scaffold the β‐sandwich, including the hotspot residues P86 and N78 [20]. Moreover, substitutions of hydrophobic amino acids by non‐hydrophobic ones may affect the binding of pVHL to the prefoldin complex, leading to protein misfolding and degradation [15].

**Table 1:** Representative disease‐associated VHL mutations and their structural and functional consequences. Information was retrieved from the VHLdb database (https://vhldb.org/mutations, accessed on 28 July 2026) according to Ref. [27] and from relevant publications.

Mutation	pVHL domain	VHL disease/tumor type	Structural effect	Functional/biological consequences	References
N78S	β domain	Type 1 VHL disease	Destabilizes β domain	Fast protein degradation; amorphous aggregates *in vitro*	[16, 20]
P86L	β domain	VHL disease Type 1; ccRCC	Destabilizes β domain	Promotes small dynamic aggregates (SDA) in *S. pombe*	[13, 17, 20]
Y98H	β domain	Type 2B VHL disease	Structurally stable	Loss‐of‐function; inhibits interaction with HIF1‐α	[14, 16]
G114R	β domain	Type 2B VHL disease	Impaired folding during *de novo* synthesis	Reduces stability of the VCB complex; impairs TRiC/CCT binding *in vitro*	[14, 24]
F119L	β domain	Type 2 VHL disease; ccRCC	Disrupts aromatic interactions	Loss of folding and function; amorphous aggregates and high aggregation propensity *in vitro*	[13, 14, 16]
F136L	β domain	Type 2B VHL disease	Disrupts aromatic interactions	Loss of folding and function; amorphous aggregates and high aggregation propensity *in vitro*	[14, 16]
P146A	β domain	ccRCC	Destabilizes pVHL structure	Promotes large slow‐moving aggregates (LSA) in *S. pombe*	[17]
I151S	β domain	ccRCC	No major structural perturbation	Impairs TRiC/CCT binding *in vitro*; reduced protein expression and inhibition of LSA formation in *S. pombe*	[17, 24]
R167W	α domain	VHL disease Type 1 and Type 2; ccRCC	Destabilizes α‐β domain interface	Impairs Elongin C interaction	[13, 14, 20]
L178P	α domain	VHL disease Type 1 and Type 2	Destabilizes α domain	Impairs Elongin C interaction	[13, 20]

Correlation between VHL mutations and protein aggregation has been studied *in vitro* [2] and in diverse systems, including bacteria [4], yeast [17], and mammalian cells [28]. Shmueli et al. [16] studied *in vitro* the effect of common oncogenic mutations on the packing of and aggregation propensity of pVHL. Two of these mutations (F119L and F136L) in the pVHL aromatic tetrahedron disrupt aromatic interactions, resulting in loss of pVHL folding, stability, and function [16]. Even though the third oncogenic mutant (N78S) was located far from this aromatic region, it also displayed increased aggregation propensity when compared to WT pVHL [16]. Conversely, the mutation Y98H was structurally stable but showed impaired HIF‐1α interaction since Y98 directly binds the hydroxyproline in HIF‐1α [16]. Overall, the mutants presented an increased hydrodynamic radius due to altered tertiary structure and compactness, which facilitates their aggregation [16]. Le Goff et al. [17] evaluated three different ccRCC‐related mutations (P86L, P146A, and I151S) located in positions critical for pVHL structural stability using a fission yeast model. Only P86L and P146A promoted aggregation; however, P86L mainly induced small aggregates, whereas P146A increased the size of large aggregates [17].

Current evidence supports that both WT and mutant pVHL possess an intrinsic propensity to misfold and populate aggregation‐prone states, with disease‐associated mutations generally increasing this tendency by reducing protein stability [16, 17]. However, most available data derive from *in vitro* assays, overexpression systems, or cellular models that may not fully reflect physiological conditions or proteostasis capacity. While these studies consistently report the formation of oligomeric or insoluble pVHL species, definitive evidence of mature amyloid fibrils in patient tissues remains limited. Accordingly, current data support an aggregation propensity rather than established pathological amyloid accumulation *in vivo*. From a mechanistic perspective, this behavior is likely influenced by cellular proteostasis networks, including Hsp70 and the TRiC/CCT chaperonin system, which buffer misfolding and restrict progression toward irreversible aggregation.

In addition to mutation‐driven destabilization, pVHL aggregation propensity has been reported to increase under cellular stress conditions, including acidosis, hypoxia, and thermal stress, which can impair proteostasis capacity [1, 4, 6]. Under such conditions, pVHL has been observed to form reversible, amyloid‐like assemblies that are often associated with stress‐responsive quality control compartments.

## 5 pVHL aggregation as a response to stressors

Under certain conditions, proteins may fail to adopt their proper functional structure, resulting in the loss or disruption of their biological functions [6, 29]. Stressors such as hypoxia, acidosis, and heat have diverse effects on the cells and may induce them to adopt either a pro‐apoptotic or a pro‐survival state, where several stress‐responsive pathways are activated in order to sustain cell viability and restore cellular homeostasis [6, 30, 31].

**Fig. 3:**
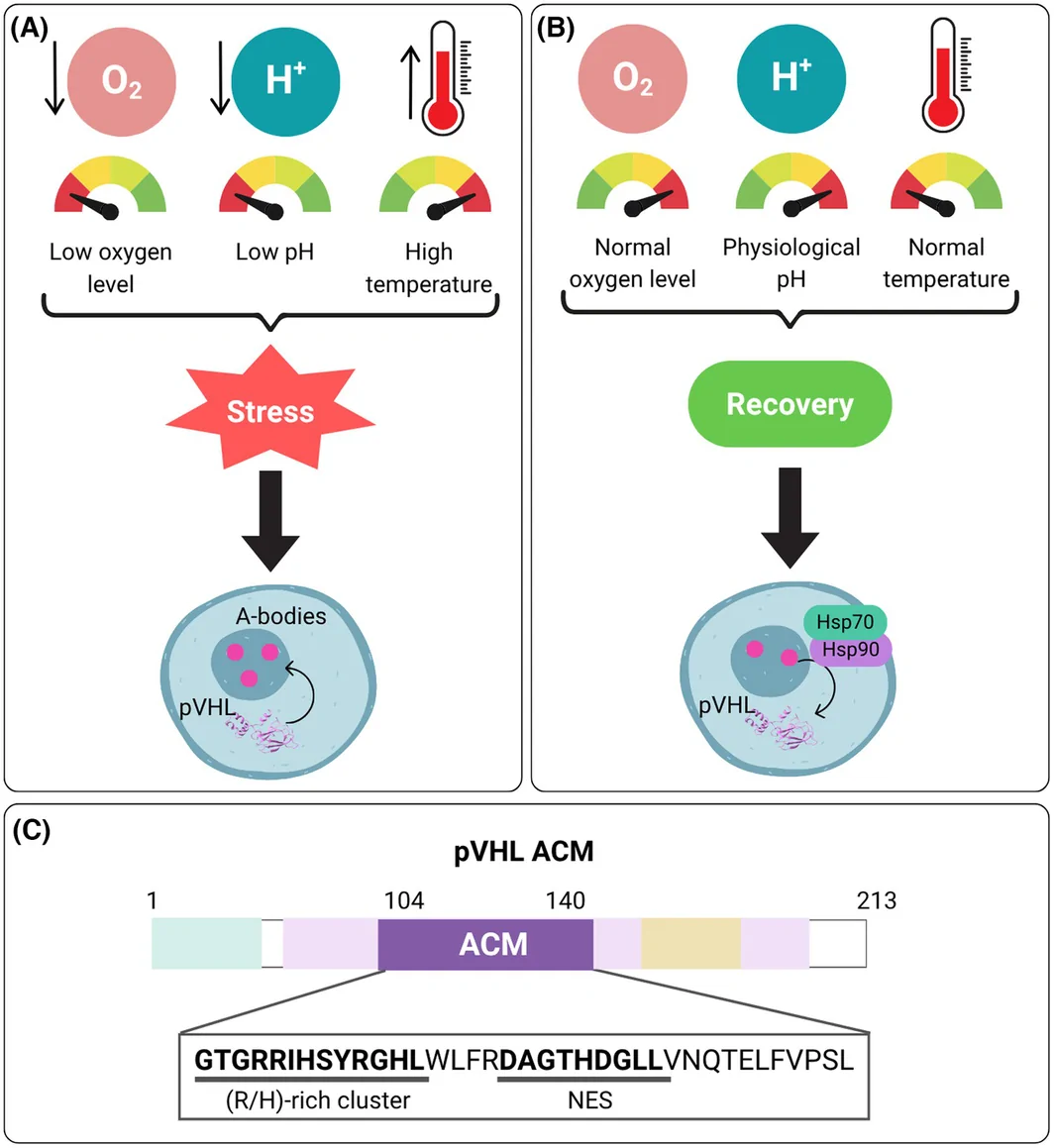
pVHL sequestration into A‐bodies under stress conditions. (A) Cellular stress conditions like hypoxia, acidosis, and heat induce protein misfolding and aggregation. During stress, reversible nuclear amyloid bodies (A‐bodies) are generated. (B) Upon stress alleviation, heat‐shock chaperones like Hsp70 and Hsp90 disassemble A‐bodies, releasing the sequestered proteins to return to their normal localization and function. (C) The amyloid‐converting motif (ACM) of pVHL comprehends the residues 104–140, which includes the nuclear export signal (NES) flanked by highly amyloidogenic sequences and an arginine/histidine rich cluster (104‐GTGRRIHSYRGHL‐116).

Hypoxia and acidosis are two important stress markers of the tumoral microenvironment, which has been demonstrated to promote misfolding and aggregation of several tumor‐associated proteins such as the mammalian target of rapamycin (mTOR), p53 and Src family kinase [4, 16, 32]. Under stress conditions, WT VHL has been detected in fibrillar structures known as amyloid bodies (A‐bodies), inducible foci that accumulate inside the nucleus (Fig. 3A) [30, 33]. These reversible aggregates are composed of heterogeneous proteins that vary significantly depending on the stressor type, which allows the cell to tailor particular stress responses by aggregating a particular subset of proteins depending on the stressor [4, 6, 34, 35]. A‐bodies are solid amyloid‐like aggregates that share biophysical properties with pathogenic amyloids, such as resistance to proteinase K digestion and staining with amyloid‐specific dyes [6]. The evolutionary conservation of A‐bodies formation across the eukaryotic domain highlights the importance of this pathway as a stress response [30, 36]. Even if aggregation renders the protein inactive, by reversible switching between mobile to static states (i.e., sequestered in A‐bodies), cells are able to regulate the protein function and response to stress conditions (Fig. 3B) [4, 8].

Proteins localizing to A‐bodies share a conserved sequence known as the “amyloid‐converting motif” (ACM), which consists of an arginine/histidine (R/H)‐rich motif adjacent to an amyloidogenic segment [5, 6]. These ACM sequences allow the interaction with ribosomal intergenic noncoding RNA (rIGSRNA), which captures and immobilizes protein in nuclear foci to regulate cellular dynamics [5, 6, 35]. In this context, the phosphoinositide 3‐kinase PI3K‐Akt signaling axis has been shown to mediate A‐body formation during stress exposure. Specifically, Akt activation represses glycogen synthase kinase‐3 (GSK3)‐dependent degradation of c‐Myc, thereby enhancing c‐Myc binding to regulatory elements of the seeding noncoding RNA and promoting the production of rIGSRNA transcripts that nucleate A‐body formation [37]. Representative ACM‐containing proteins include CDK1, HAT1, HDAC2, and ApoE, supporting the notion that this mechanism is broadly conserved across unrelated functional classes [5]. In pVHL, the ACM comprehends the residues 104–140, which includes the nuclear export signal (NES) flanked by highly amyloidogenic sequences and an arginine/histidine‐rich cluster (^104^GTGRRIHSYRGHL^116^) (Fig. 3A) [4, 6, 35]. This bipartite sequence is capable of recruiting the pVHL104‐140 peptide to A‐bodies under acidic conditions [4, 6, 35]. This sequestration prevents pVHL from targeting HIF‐α for degradation, allowing the transcription factors to accumulate and activate the expression of genes involved in oxygen homeostasis and stress adaptation [38, 39]. Mechanistically, aggregation may mask the nuclear export signal, thereby disrupting pVHL shuttling between the cytoplasm and nucleus, which is essential for its E3 ligase activity [4, 8, 35]. Moreover, Mekhail and colleagues [8] also found that Cullin2, another component of the VCB/Cul‐2 E3 ligase complex, is sequestered to the A‐bodies by associating with pVHL. These data suggest that pVHL may regulate the ubiquitination machinery by modulating the dynamic states of its components in response to acidic pH [8].

Physiological A‐bodies are characterized by their reversibility once the stress is eliminated. When standard growth conditions are restored (Fig. 3C), the heat‐shock chaperone machinery, including members such as Hsp70 and Hsp90, disassembles amyloid bodies and pVHL is released from the insoluble fraction ready to return to its original localization, without undergoing degradation, and restoring its ability to degrade HIF‐α [1, 5, 6, 8]. Thus, physiological amyloidogenesis acts as a regulatory mechanism that negatively controls protein activity without degradation while preventing protein damage due to stringent conditions [6, 39].

## 6 Amyloidosis: Role in cancer and cell dormancy

Amyloidosis has been reported as a physiological mechanism adopted by many mammalian cells in response to stressors [5]. Consistent with this response, the formation of A‐bodies has been associated with the induction of a dormant cellular state [5, 6, 40]. Cell dormancy is a reversible state in which cells remain metabolically active but are temporarily arrested in terms of cell division and proliferation [5]. The correlation between A‐body formation and cell dormancy has been investigated in particular in breast and prostate cancer cells [5]. By entering into a dormant state, cells are able to store large amounts of proteins while still remaining viable during extended periods of time [5]. In this manner, cells can adapt and survive prolonged periods of extracellular stress, thereby contributing to tumor progression [4, 41]. Several survival‐signaling pathways have been implicated in the regulation of cellular dormancy, including Wnt and Notch, the PI3K‐Akt pathway, the cell cycle regulators p130 and p27 (KIP1), EGF receptor signaling, mTOR‐dependent activation of GTPases, as well as pathways controlling apoptosis, autophagy, and oxidative stress responses [5, 9]. Hypoxic conditions characteristic of many solid tumors stabilize HIF‐α, thereby activating transcriptional programs that promote cellular adaptation and more aggressive tumor phenotypes [42]. In mammary cancer cells, HIF‐α overexpression has been associated with tumor aggressiveness, resistance to chemotherapy, and radiotherapy [41, 42, 43]. These observations highlight the importance of the VHL/HIF‐α regulatory axis in linking hypoxic stress to the induction of tumor cell dormancy and therapy resistance.

**Fig. 4:**
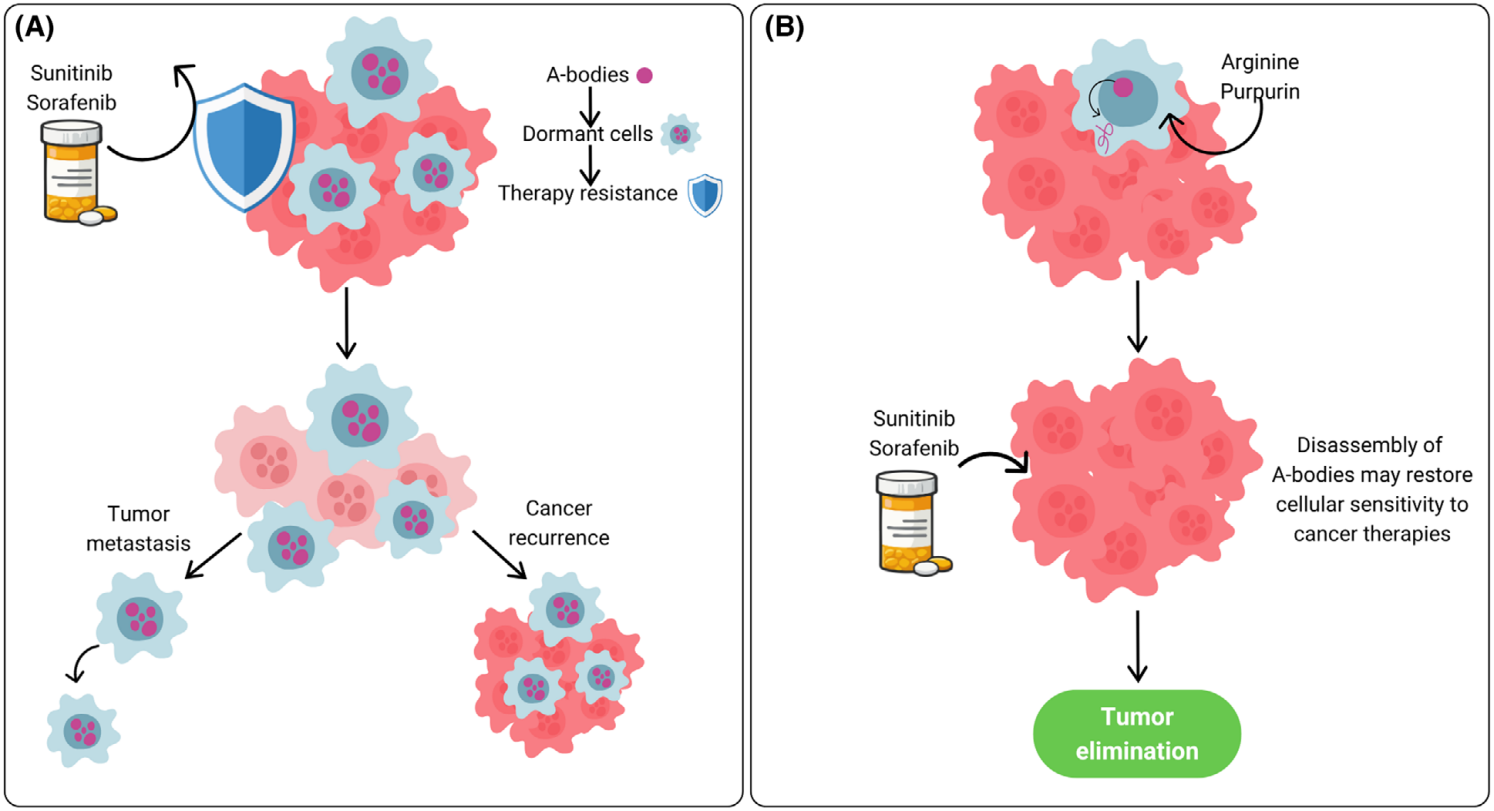
A‐body formation promotes cell dormancy. (A) Cell dormancy leads to resistance to traditional cancer therapies, which ultimately contributes to tumor metastasis and cancer recurrence. (B) Small molecules that can prevent aggregation such as arginine and purpurin could restore protein function and potentially *awaken* dormant cells to resensitize them to traditional therapies.

Despite its benefits for cell survival, dormancy negatively affects cancer therapy efficacy since most of currently approved drugs primarily target highly proliferative cancer cells [9, 44]. Dormant cancer cells can evade these treatments, serving as a reservoir of therapy‐resistant cells that may later re‐enter the cell cycle and drive tumor recurrence and metastasis (Fig. 4A) [4, 38, 40]. In this context, A‐body formation contributes to the establishment and maintenance of the dormant state under stress conditions, suggesting that these structures could represent a potential target for therapeutic intervention [5]. There are different perspectives on how to address A‐body formation and dormancy in order to improve available cancer therapies and develop novel alternatives [38]. On the one hand, preventing A‐body disaggregation could maintain tumor cells in a non‐proliferative state for prolonged periods [5]. Thus, rather than attempting to eliminate dormant tumor cells, an approach that may promote more aggressive tumor behavior and whose clinical efficacy remains uncertain, the focus could be placed on reinforcing and maintaining the dormant state [5, 9]. However, such an approach would likely require continuous treatment, potentially leading to toxicity and poor patient compliance [9]. Alternatively, another strategy could involve preventing cells from entering dormancy or awakening dormant cells in a controlled manner, thereby enabling their elimination with currently available therapies that target proliferating cells (Fig. 4B) [9]. Since A‐bodies promote cell dormancy and pVHL is one of their main constituents, preventing the formation of these structures could represent a novel strategy to counteract therapy resistance in cancer cells [4, 5, 45].

## 7 Current therapies, limitations and dormancy

Available therapies for VHL‐related tumors rely primarily on surgery; however, recurrence is common even after tumor removal [25, 46]. First‐line treatment for patients with renal cell carcinoma (RCC) is sunitinib, a multitargeted tyrosine kinase inhibitor (TKI) that inhibits the VEGF receptor [47]. Another TKI, sorafenib, is approved for patients with advanced RCC [47]. In contrast to TKIs, temsirolimus acts through a different mechanism by inhibiting mTOR, leading to G1 cell cycle arrest and suppression of tumor angiogenesis [47]. Although these therapies significantly improved the prognosis of RCC, they still face limited efficacy, heterogeneous outcomes, and drug resistance in subsets of patients [13, 47].

Chittiboina et al. [10] demonstrated that vorinostat reduced pro‐tumorigenic features in central nervous system hemangioblastomas patients with germline missense VHL mutations, where pVHL maintains its intrinsic functions but is rapidly degraded. Short‐term daily administration of this drug demonstrated biological activity within VHL‐associated tumors by markedly increasing pVHL expression in neoplastic stromal cells, leading to suppression of key HIF signaling pathway genes [10]. Mechanistically, vorinostat reduces the interaction between Hsp90 and pVHL, modulating the proteasomal pathway and blocking the degradation of pVHL, which retains sufficient tumor suppressor activity [10]. Future research is needed to determine the long‐term impact of this treatment on controlling VHL‐associated tumors, including hemangioblastomas, renal cell carcinoma, pheochromocytomas, and pancreatic neuroendocrine tumors [10].

One of the main limitations of current cancer therapies is the above‐mentioned cell dormancy, which has been related to cancer relapse and more aggressive tumors, as well as poor efficacy of cancer treatments (Fig. 4A) [9]. Conventional cancer therapies target highly proliferative and fast‐dividing cells, which explains why they often fail in eradicating dormant cancer cells [9]. An example has been demonstrated with paclitaxel therapy, which blocks mitosis and leads to cell death. In this case, the sensitivity toward the drug increased when quiescent fibroblasts resumed their proliferation [9]. Interestingly, the response of dormant cells to cancer therapies is also dependent on the type of tumor and location of dormant cells [9]. Alkylating agents exert their cytotoxic activity by transferring alkyl groups to DNA bases, generating DNA adducts and cross‐links that impair DNA replication and transcription and ultimately trigger cell death. The alkylating agent nimustine has shown effective targeting of nonproliferating cells in a mouse model BRCA‐ and p53‐deficient, while melphalan, another alkylating agent, was not effective against dormant cells in a preclinical model for myeloma [9]. One strategy to prevent tumor recurrence resulting from dormant cell reactivation is to maintain cells in a dormant state [9]. So far, several drugs inhibiting growth signals or targeting the tumor microenvironment have been proposed to maintain dormancy, as well as inhibition of cell cycle regulators [9]. On the other hand, awakening dormant cells could sensitize them to conventional chemotherapeutics, as successfully seen in cases of hematologic cancer, whereas only a few studies have explored the reactivation of dormant cells in solid tumors [9]. Although this approach requires extreme caution to prevent more aggressive tumors and metastasis, reactivating dormant cells has shown to be effective for some particular tumors in preclinical models and present as a novel clinical strategy [9].

## 8 pVHL amyloid formation is inhibited by small molecules

The discovery of pVHL amyloidogenesis and its possible correlation with cell dormancy presents new therapeutic possibilities to explore, since no drug directly targeting pVHL aggregation has yet been developed [4]. Osmolytes are known to prevent protein aggregation and help with protein refolding [16, 48]. Shmueli et al. [16] tested three different classes of osmolytes (amino acids, polyols, and methylamines) to determine the most effective in preventing pVHL (WT and mutants) aggregation. The different osmolytes were evaluated using a pVHL‐EGFP reporting system and bacterial aggregation assays [16]. The most effective osmolyte in restoring pVHL folding and inhibition of aggregation was arginine [16]. This amino acid has long been used experimentally to inhibit protein aggregation [49]. Arginine acts by binding to anionic side chains via H‐bond‐reinforced ionic contacts, which hampers the formation of protein–protein salt bridges [49]. This effect on protein aggregation was comparable to that of the free guanidinium moiety, which has been pointed out as the group responsible for arginine's effect on protein stability and refolding [49]. Arginine not only mediates protein refolding but also helps in the restoration of pVHL function and its ability to interact with substrates such as HIF‐α, which has been demonstrated in *Drosophila* and human renal cell carcinoma (RCC) cells [48]. Thus, it has been proposed as a chemical chaperone to increase WT and mutant pVHL solubility, restoring the functionality of the pVHL/HIF‐α pathway [48]. Considering that arginine is a naturally occurring amino acid that is widely available, it represents a promising candidate for the treatment of VHL syndrome and VHL‐associated tumors [4, 48]. Despite these advantages, its low efficacy and bioavailability have limited arginine's application as a therapeutic [4]. This limitation prompted the search for alternative aggregation‐modulating compounds. Purpurin, a bona fide amyloid inhibitor, has been shown to inhibit *in vitro* fibrillation of the pVHL104‐140 peptide in a concentration‐dependent manner, raising the potential for restoring pVHL function and sensitizing dormant tumor cells to already existing cancer treatments [4]. These findings suggest a novel therapeutic avenue for ccRCC by focusing specifically on reversing pVHL aggregation [4, 17, 50].

## 9 Potential role of pVHL amyloid sequestration in cancer aggressiveness and dormancy

The *VHL* gene appears mutated or deleted in 80% of sporadic ccRCC [2, 13]; however, tumors expressing WT pVHL can also exhibit aggressive and metastatic behavior [34, 51, 52]. Some studies have hypothesized that aggregation of WT pVHL may play a role in ccRCC aggressiveness [4, 33]. The harsh conditions of the tumor microenvironment, including hypoxia, elevated temperature, acidosis, and other cellular stressors, promote protein aggregation, leading to pVHL sequestration and a functional loss‐of‐function state even in the absence of VHL mutations [4, 32].

The convergence of recent findings presents an intriguing hypothesis: could pVHL sequestration into amyloid‐like aggregates mimic a loss‐of‐function state and represent a mechanism underlying cancer aggressiveness and cell dormancy? This proposition emerges from several key observations across the literature. On the one side, pVHL sequestration in amyloid bodies could mimic a loss‐of‐function state leading to HIF‐α accumulation even under normoxic conditions, promoting a pseudo‐hypoxic phenotype. Msaki et al. [53] observed that dormant disseminated tumor cells exhibit hypoxic transcriptional signatures, suggesting that sustained HIF‐α activation might promote cell dormancy, rather than tumor growth under particular contexts [11, 53]. Moreover, pVHL engages in dynamic nucleocytoplasmic shuttling, which is crucial for its regulatory functions and interaction with the ECV complex [4]. Amyloid sequestration disrupts pVHL's shuttling capacity and immobilizes it into the nucleolar compartment, which could impair its function and trigger cellular stress responses that favor dormancy [4, 8].

Besides its canonical function in HIF‐α regulation, pVHL also has hypoxia‐independent functions that may provide additional context for understanding how pVHL dysfunction influences tumor progression and cell dormancy [12, 54]. In particular, pVHL has been implicated in the regulation of cell‐cycle checkpoints and cellular senescence through oxygen‐independent mechanisms, expanding the spectrum of pVHL functions beyond HIF regulation [55, 56]. In addition, the pVHL β‐domain interacts with and inhibits members of the protein kinase C (PCK) family, regulating cellular functions like angiogenesis, proliferation, differentiation, and cell survival [54]. Similarly, pVHL also inhibits the Akt1 isoform from the Akt kinase family, involved in metabolism, proliferation, cell survival, growth, and angiogenesis [54]. Many other pathways have been functionally associated with pVHL, positioning it as a critical player for maintaining proper cellular signaling cascades correlated to cell growth, proliferation, and metabolism. Mizejewski et al. [5] note that amyloidosis naturally induces cell growth cessation and proliferation arrest, suggesting that a cellular response to signaling disruption is to enter a protective dormant state. The convergence of these impaired functions could lead to a unique cellular state: cells experience *pseudo‐hypoxia* (due to HIF‐α accumulation), disrupted signaling (because of kinase dysregulation), compromised spatial organization (due to lost shuttling), and widespread protein interaction deficits. The mechanistic link between pVHL aggregation and the induction of a dormant cellular state remains largely unexplored. Elucidating this relationship may provide new insights into the molecular basis of cancer dormancy and therapeutic resistance.

## 10 Conclusions

The tumor suppressor pVHL is an intrinsically unstable, molten‐globule–like protein whose folding depends on chaperones and proteostasis [22]. Its structural vulnerability makes it sensitive to destabilizing conditions, suggesting that clear cell renal cell carcinoma, and potentially other VHL‐associated tumors, may result not only from VHL mutations but also from reversible, stress‐induced loss of pVHL function [4, 6, 35].

Although direct evidence of pVHL aggregates in tumor tissues is still lacking, several pathogenic variants display increased aggregation propensity *in vitro* and in cellular systems, suggesting that mutation‐driven amyloidogenesis may contribute to pVHL functional inactivation [2, 17]. Similar aggregation phenomena have been observed for other tumor suppressors, such as p53 and PTEN, whose amyloid‐like assemblies impair activity and can exert dominant‐negative effects; whether comparable mechanisms occur in pVHL remains to be determined [3, 32].

Cellular stress represents another condition that can promote reversible WT pVHL aggregation. Under severe stress, pVHL can be transiently sequestered into amyloid bodies (Fig. 3), nuclear assemblies associated with reduced cellular activity and entry into dormancy [4, 5, 6, 8]. The functional consequences of this sequestration are best investigated in the hypoxia pathway: pVHL inactivation stabilizes HIF‐α and activates transcriptional programs that promote oxygen homeostasis and metabolic adaptation [12, 42, 54]. In contrast, the impact of pVHL aggregation on its HIF‐independent functions, such as regulation of apoptosis and cell‐cycle progression, partly mediated by p53, remains largely unexplored.

Finally, mutations in the VHL gene may influence not only protein stability but also the dynamics and reversibility of these assemblies, potentially prolonging pVHL inactivation during cellular stress. Future studies investigating how specific mutations influence the formation and reversibility of pVHL aggregates, particularly within A‐bodies, will be important to clarify the role of these assemblies in regulating both HIF‐dependent and HIF‐independent pathways and in promoting cellular adaptation or dormancy in tumors.

From a therapeutic perspective, understanding pVHL aggregation and cell dormancy may open new avenues for the treatment of clear cell renal cell carcinoma and VHL‐associated tumors. This emerging field is beginning to explore strategies aimed at restoring pVHL function by stabilizing its fold or preventing its sequestration into amyloid assemblies [4, 29, 48, 57]. For instance, the chemical chaperone arginine and amyloid inhibitors such as purpurin have been proposed as aggregation‐modulating molecules capable of reducing pVHL aggregation and potentially sensitizing dormant or drug‐resistant tumor cells to existing therapies, although their clinical efficacy remains untested [4, 29, 48].

Together, these perspectives position pVHL at the intersection between protein aggregation biology and cancer research. A deeper understanding of how pVHL aggregation and A‐body formation influence cell dormancy and therapy resistance could uncover previously unrecognized regulatory mechanisms and support the development of more effective therapeutic strategies.

## Conflict of interest

The authors declare no conflicts of interest.

## Author contributions

LA wrote the draft; EL and ST conceptualized the review article and critically revised the manuscript.

## Data Availability

This article is a review and does not contain original data generated by the authors. Therefore, no datasets were deposited in public repositories.
